# The effect of sustained-release CARvedilol in patients with hypErtension and heart failure with preserved ejection fraction: a study protocol for a pilot randomized controlled trial (CARE-preserved HF)

**DOI:** 10.3389/fcvm.2024.1375003

**Published:** 2024-04-26

**Authors:** Minjae Yoon, Sung-Ji Park, Byung-Su Yoo, Dong-Ju Choi

**Affiliations:** ^1^ Division of Cardiology, Department of Internal Medicine, Seoul National University Bundang Hospital, Seongnam, Republic of Korea; ^2^ Division of Cardiology, Department of Internal Medicine, Cardiovascular Imaging Center, Heart Vascular Stroke Institute, Samsung Medical Center, Sungkyunkwan University School of Medicine, Seoul, Republic of Korea; ^3^ Department of Internal Medicine, Yonsei University Wonju College of Medicine, Wonju, Republic of Korea

**Keywords:** beta-blocker, heart failure with preserved ejection fraction, hypertension, sustained-release carvedilol, global longitudinal strain

## Abstract

**Background:**

Although beta-blockers improve clinical outcomes in heart failure with reduced ejection fraction, the benefit of beta-blockers in heart failure with preserved ejection fraction (HFpEF) is uncertain. Global longitudinal strain (GLS) is a robust predictor of heart failure outcomes, and recent studies have shown that beta-blockers are associated with improved survival in those with low GLS (GLS <14%) but not in those with GLS ≥14% among patients with LVEF ≥40%. Therefore, the objective of this trial is to evaluate the effect of sustained-release carvedilol (carvedilol-SR) on the outcome [N-terminal pro-B-natriuretic peptide (NT-proBNP) concentration] in patients with hypertension and HFpEF and will assess the differential effects of these drugs on the outcome, according to the GLS categories.

**Methods:**

This prospective randomized double-blind multicenter trial (CARE-preserved HF) will include 100 patients with HFpEF from three tertiary hospitals in South Korea. Patients with HFpEF and hypertension aged ≥20 years who have evidence of functional and structural heart disease on echocardiography and elevated natriuretic peptide will be enrolled. Eligible participants will be randomized 1:1 to either the carvedilol-SR group (*n* = 50) or the placebo group (*n* = 50). Patients in the carvedilol-SR group will receive 8, 16, 32, or 64 mg carvedilol-SR once daily for 6 months, and the dose of carvedilol will be up-titrated at the discretion of the treating physicians. The primary efficacy outcome was the time-averaged proportional change in N-terminal pro-B-natriuretic peptide concentration from baseline to months 3 and 6. We will also evaluate the differential effects of carvedilol-SR on primary outcomes according to GLS, using a cut-off of 14% or the median value.

**Discussion:**

This randomized controlled trial will investigate the efficacy and safety of carvedilol-SR in patients with HFpEF and hypertension.

**Clinical Trial Registration:**

ClinicalTrial.gov, identifier NCT05553314.

## Introduction

The prevalence of heart failure (HF) with preserved ejection fraction (HFpEF) is increasing (1–3). Approximately 50% of all HF patients have HFpEF, and in the general population aged ≥60 years, over 4% were identified with HFpEF (4–7). Beta-blockers are known to reduce mortality in HF with reduced ejection fraction (HFrEF) (8) and are recommended by current guidelines (9–11). However, the effects of beta-blockers on HFpEF remain controversial. The SENIORS randomized controlled trial, examining the effects of nebivolol on mortality and cardiovascular admissions, revealed an overall benefit in patients with an left ventricular ejection fraction (LVEF) >35% (12); however, the study had insufficient patients and events to draw any conclusions in those with a more preserved LVEF (LVEF ≥50%). In previous randomized clinical trials and meta-analyses, the effect of beta-blockers was not consistent in patients with HFpEF (13–16). Also, in many real-world cohorts, the use of beta-blockers in patients with HFpEF is not associated with improved clinical outcomes of HF (17, 18). Although a recent meta-analysis including 16 randomized trials or observational cohort studies showed that beta-blocker therapy reduces all-cause mortality in patients with HFpEF (15), the benefit of beta-blockers in HFpEF is considered unclear. In addition, a *post hoc* analysis of the TOPCAT trial showed that beta-blockers were associated with an increased risk of hospitalization for HF in patients with HFpEF (19). However, in real-world clinical practice or recent trials, a large proportion of patients with HFpEF are currently receiving beta-blockers (19–22), reflecting the potential benefits of beta-blockers in HFpEF through sympathetic antagonism, including lower blood pressure (BP) and heart rate (HR) and prolong diastolic filling time (16, 23).

Myocardial strain is based on the speckle-tracking method and is an emerging parameter for evaluating the systolic function of the heart in a more sophisticated manner than conventional methods (24, 25). In a recent study, Park et al. showed that left ventricular (LV) global longitudinal strain (GLS) was a better predictor of clinical outcomes than LVEF and that patients with similar GLS had similar prognoses, regardless of LVEF (26). Moreover, a significant proportion of patients with HFpEF have low GLS despite preserved LVEF (27). Recently, in a retrospective study of 1,969 patients with HF and LVEF of ≥40%, beta-blockers were associated with improved survival in those with low GLS (GLS <14%) but not in those with GLS ≥14% (28).

Because HFpEF is a heterogeneous and complex syndrome rather than a single disease entity (7), we hypothesized that GLS may be a factor determining the effects of beta-blockers in HFpEF. Carvedilol is a non-selective beta-blocker that has been extensively studied in patients with HFrEF (8, 29). Recently, sustained-release carvedilol (carvedilol-SR) has been developed, which can maintain an effective plasma concentration without exceeding the critical level for adverse effects (30). Carvedilol-SR has the advantage of being administered only once a day compared with the twice-daily administration of immediate-release carvedilol (carvedilol-IR). Therefore, this trial aim to evaluate the efficacy of carvedilol-SR on the outcome [N-terminal pro-B-natriuretic peptide (NT-proBNP) concentration] in patients with hypertension and HFpEF, and will assess the differential effects of these drugs on the outcome, according to GLS categories.

## Methods and analysis

### Study design

**Figure 1:**
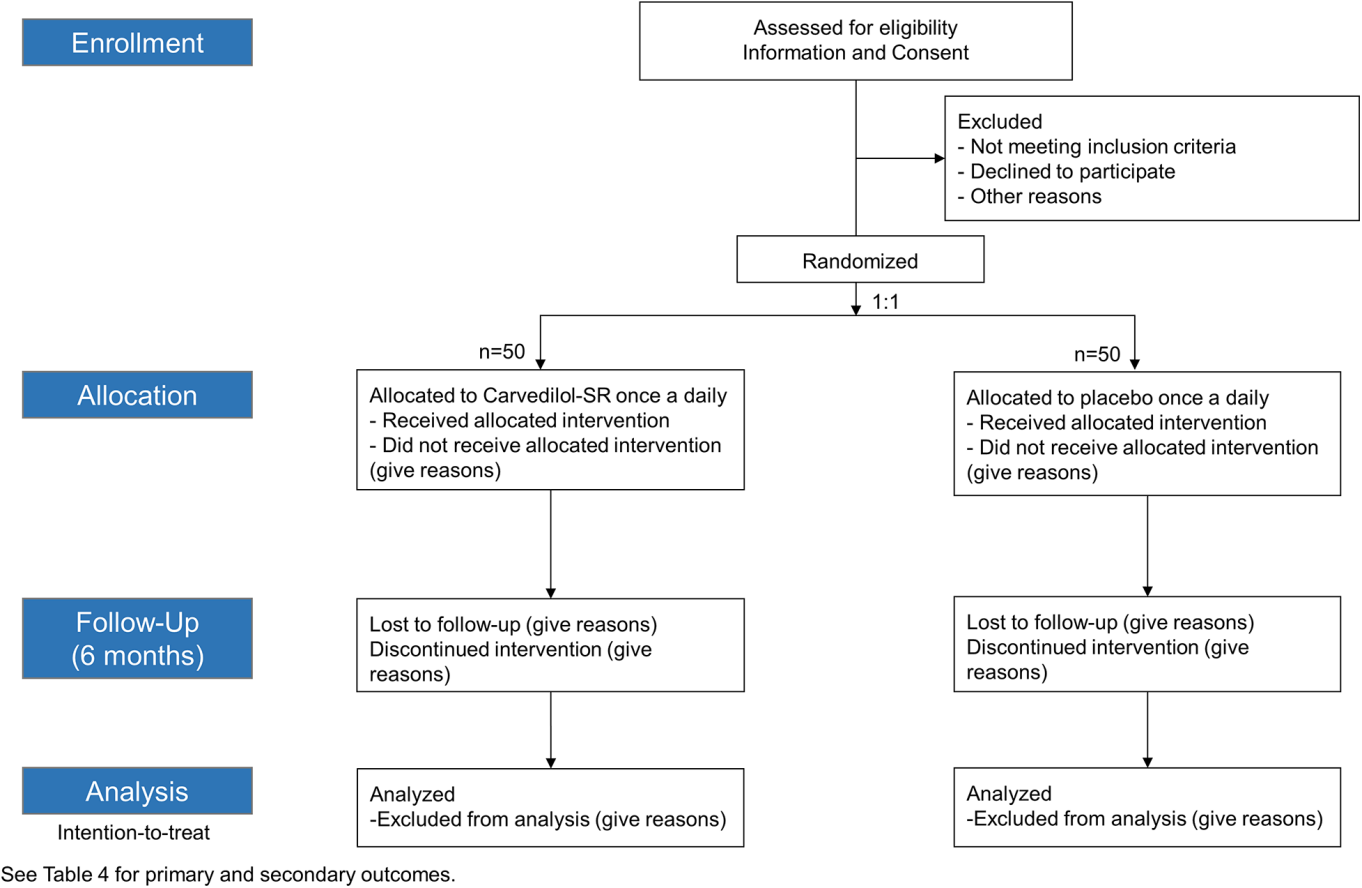
Study flowchart. Carvedilol-SR, sustained-release carvedilol.

Sustained-release CARvedilol in patients with hypErtension and Heart failure with Preserved ejection fraction (CARE-preserved HF) is a multicenter, prospective, randomized, double blinded, phase 4 pilot study that assesses the efficacy of carvedilol-SR compared with placebo in patients with hypertension and HFpEF. The study flow chart is presented in Figure 1. Three tertiary university hospitals in South Korea participated in the study. Enrollment began in November 2021, is ongoing and is expected to be completed by early 2024. The trial design was registered at ClinicalTrial.gov (NCT05553314).

### Study population

We have been enrolling patients with hypertension and HFpEF aged ≥20 years. Patients must have symptoms of HF and elevated natriuretic peptides at the screening visit: NT-proBNP ≥220 pg/ml [or B-type natriuretic peptide (BNP) ≥80 pg/ml] for patients with sinus rhythm (SR), or ≥660 pg/ml (or BNP ≥240 pg/ml) for patients with atrial fibrillation (AF). For the hypertension criteria: (1) patients' average systolic blood pressure (SBP) must be ≥140 mmHg or diastolic blood pressure ≥90 mmHg, obtained from BP measured three times on the reference arm in the sitting position at screening; (2) for those taking anti-hypertensive medications who are on stable antihypertensive dosage that has not been adjusted for 8 weeks, the SBP must be ≥110 mmHg. Patients with SBP <110 mmHg or resting HR <60 beats/min at the screening visit will be excluded. For the HFpEF criteria, we referred to the consensus recommendation of the Heart Failure Association of the European Society of Cardiology (31). LVEF should be ≥50% on echocardiography within 2 months before randomization. In addition, patients should have one or both of the following evidence of structural heart disease: (a) left atrial volume index ≥29 ml/m^2^ in patients with SR or ≥34 ml/m^2^ in patients with AF, (b) left ventricular mass index ≥115 g/m^2^ in males or ≥95 g/m^2^ in females. Patients should also have at least one indication of functional heart disease in terms of average E/e', septal and lateral e', peak tricuspid regurgitation velocity, and pulmonary artery systolic pressure. Patients will be excluded if they are taking any beta-blockers within 4 weeks prior to enrollment or if they have a contraindication to beta-blockers. Patients with severe chronic kidney disease (creatinine >2.4 mg/dl) or liver enzyme levels three times the normal upper limit will be excluded. Detailed inclusion and exclusion criteria are shown in Table 1.

**Table 1:** Inclusion and exclusion criteria.

Inclusion criteria
1. ≥20 years of age, male or female 2. Patients with an SBP of ≥140 mmHg or a DBP of ≥90 mmHg, or if taking anti-hypertensive medication, an SBP of ≥110 mmHg. 3. Current symptom(s) of HF at screening visit^a^ 4. Elevated natriuretic peptides: NT-proBNP ≥220 pg/ml (BNP ≥80 pg/ml) for patients with SR or ≥660 pg/ml (BNP ≥240 pg/ml) for patients with AF at screening visit 5. LVEF ≥50% by echocardiography within 2 months before the randomization 6. Structural heart disease evidenced by one or both of the following echocardiography findings a) LAVI ≥29 ml/m^2^ in patients with SR or ≥34 ml/m^2^ in patients with AF b) LVMI ≥115 g/m^2^ in male or ≥95 g/m^2^ in female 7. Functional heart disease evidenced by at least one of the following echocardiography findings a) Average E/e’ ≥9 b) Septal e’ <7 cm/s c) Lateral e’ <10 cm/s d) Peak tricuspid regurgitation velocity >2.8 m/s e) Pulmonary artery systolic pressure >35 mmHg
Exclusion criteria
1. SBP <110 mmHg, or resting heart rate <60 beats/min at the screening visit 2. Patients who have a contra-indication to beta-blockers (including those with second-or third-degree atrioventricular block; respiratory diseases, including severe or uncontrolled asthma, chronic obstructive lung disease, allergic rhinitis or glottis edema; or severe peripheral vascular disease [Raynaud's syndrome, intermittent claudication]) 3. Patients who take any beta-blockers within 4 weeks before enrollment or who are expected to take another beta-blocker after randomization 4. Patients with a history of the following diseases - Amyloidosis, hypertrophic cardiomyopathy with left ventricular outflow tract obstruction, severe aortic or mitral valve stenosis, acute coronary syndrome within 3 months, percutaneous coronary intervention within 3 months, and recent cardiothoracic surgery within 3 months - Severe cerebrovascular events including ischemic stroke and hemorrhagic stroke within 6 months 5. Patients with significant kidney or liver disease - Creatinine >2.4 mg/dl or - Alanine transaminase or aspartate transaminase level is at least 3 times higher than the normal upper limit 6. Patients with a history of secondary hypertension or suspected secondary hypertension 7. Moderate-to-severe retinopathy (for example, retinal hemorrhage, visual disturbance, retinal microaneurysm within 6 months) 8. Patients in a clinical status that can significantly influence absorption, distribution, metabolism, and secretion of drugs for clinical trials: a. History of major gastrointestinal surgery, such as gastrectomy or gastric bypass surgery b. Inflammatory bowel disease within 12 months c. Current gastric ulcer, pancreatic function abnormality including pancreatitis, gastrointestinal/rectal bleeding which requires treatment d. Current urologic stenosis or obstruction which requires treatment 9. Chronic inflammatory diseases which require long-term use of anti-inflammatory treatment 10. Hypersensitivity to carvedilol 11. Patients with malignant disease including lymphoma and leukemia within 5 years 12. Patients who were prescribed other medication for any other clinical trials within the pre-analytical 28 days 13. Patients who are expected to have prolonged hospital stay due to other medical problems other than chronic heart failure (for example, femoral neck fracture) 14. Patients with a history of alcohol or drug abuse within the last 12 months 15. Patients with potential pregnancy or breastfeeding 16. Patients who are considered inappropriate by researchers to participate in the clinical trial

AF, atrial fibrillation; BNP, B-type natriuretic peptide; DBP, diastolic blood pressure; GLS, global longitudinal strain; HF, heart failure; LAVI, left atrial volume index; LVMI, left ventricular mass index; NT-proBNP, N-terminal pro B-type natriuretic peptide; SBP, systolic blood pressure; SR, sinus rhythm.

^a^ HF symptoms include dyspnea on exertion, paroxysmal nocturnal dyspnea, orthopnea, or leg swelling.

### Patient recruitment and randomization

**Figure 2:**
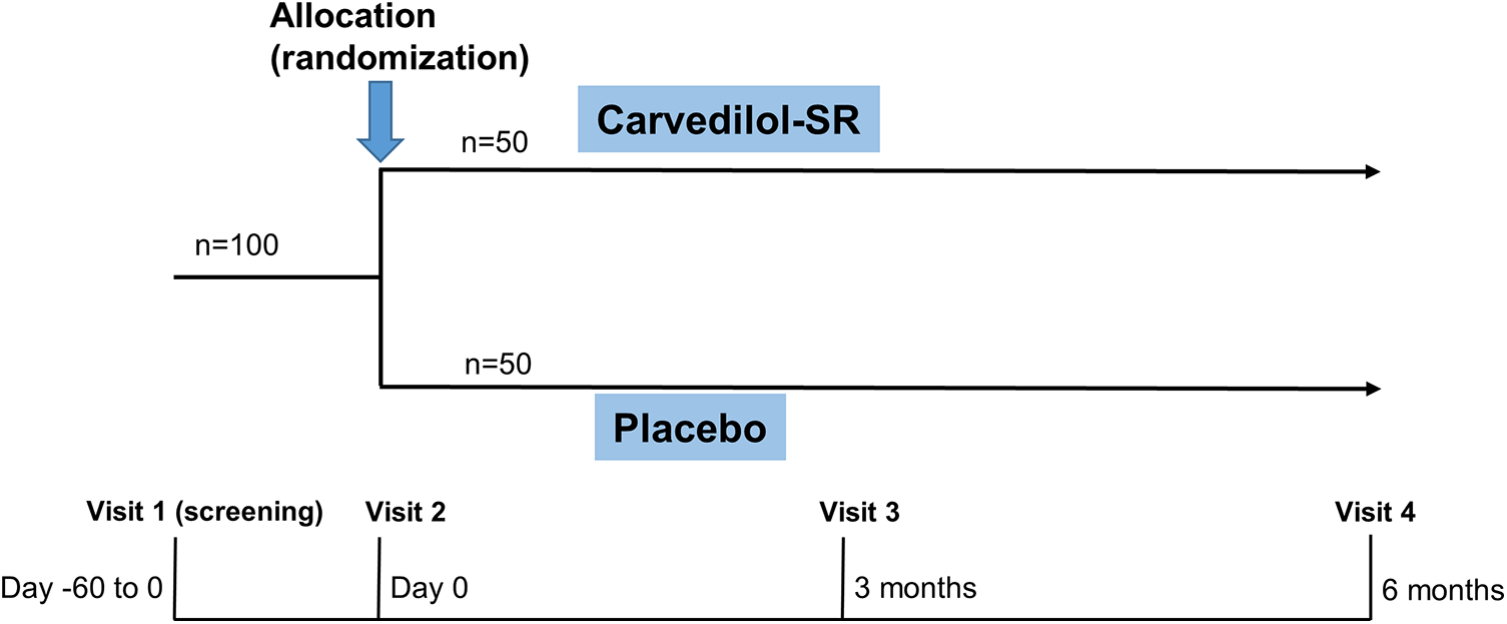
Study design of CARE-preserved HF. Patients will be randomized to either carvedilol-SR group or placebo groups. Carvedilol-SR, sustained-release carvedilol.

Potential participants will be screened (visit 1) on an outpatient or inpatient basis (Figure 2). After a comprehensive interview, eligible participants will be asked to provide written informed consent to participate in the trial. A baseline survey of eligible participants covering demographics and cardiovascular comorbidities will be conducted by research nurses (Table 2). Then, using a web-based central randomization service (http://matrixmdr.com), eligible participants will then be randomly assigned to either the carvedilol-SR group or the placebo group in a 1:1 ratio (visit 2). The randomization block will be generated by an independent statistician unrelated to this study using the SAS randomization program.

**Table 2:** Standard protocol items: recommendation for interventional trials (SPIRIT) checklist.

	Study period			
	Enrolment (screening)	Allocation	Post-allocation	Close-out
Timepoint	Visit 1 (day −60–0)	Visit 2 (day 0)	Visit 2 (3 months)	Visit 3 (6 months)
Enrolment				
Eligibility screen	X			
Informed consent	X			
Allocation		X		
Interventions				
Intervention		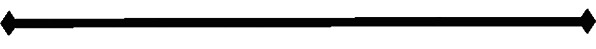		
Control		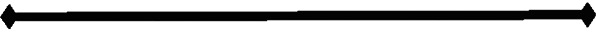		
Assessments				
Baseline characteristics	X			
NT-proBNP	X	X		X
Echocardiography	X			X
12 lead electrocardiogram	X			
Vital signs	X	X	X	X
Secondary endpoints		X	X	X

NT-proBNP, N-terminal pro B-type natriuretic peptide.

### Intervention and follow-up

After randomization, patients in the carvedilol-SR group will receive carvedilol-SR 8, 16, 32, or 64 mg once daily for 6 months. Carvedilol-SR (trade name: Dilatrend SR) was manufactured and distributed by Chong Kun Dang Pharmaceutical Corporation in South Korea. The carvedilol dose will be up-titrated at the discretion of the treating physician. The use of beta-blockers other than carvedilol will not be allowed during the study. To avoid drug-drug interactions that may cause confounding effects, drugs with potential pharmacodynamic and pharmacokinetic interference will be prohibited during clinical trials (Table 3).

**Table 3:** Drugs contraindicated during this clinical trial.

Contraindication details
1. Other beta-blockers except the study medication 2. Steroid medication. Locally applied drugs are allowed. 3. Estrogen medications. If necessary, low-dose hormonal therapy can be continued for therapeutic purpose at the same dose during the study period 4. Sympathomimetic drugs such as catecholamines 5. Anti-psychotics, monoamine oxidase inhibitors, lithium, anti-depressants Sedatives and anti-anxiolytics (benzodiazepines and their antagonists, barbiturates, hypnotics such as zolpidem) can be intermittently used according to the physician's decision, while their usage is absolutely prohibited 7 days before the scheduled visits 6. Immunosuppressants 7. Thyroid hormones. If necessary, thyroid hormonal therapy can be prescribed for therapeutic purpose at the same dose during the study period

Non-dihydropyridine calcium channel blockers and antiarrhythmics should be used with caution.

Follow-up visits will be scheduled at 3 (visit 3) and 6 (visit 4) months after randomization (Figure 2 and Table 2). Each clinic visit comprises history-taking, physical examination, laboratory tests, and echocardiography. Drug compliance, adverse effects, and the use of additional concomitant drugs will also be checked. Unscheduled visits will be recorded.

### Study outcomes and variables

The outcomes of this study are presented in Table 4. The primary efficacy outcome will be the time-averaged proportional change in NT-proBNP concentration from baseline through months 3 and 6. The secondary endpoints included the proportion of patients with an NT-proBNP decrease >20% from baseline through months 3 and 6, composite of all-cause mortality and all-cause hospitalization at 6 months. We will also assess changes in BP, HR, New York Heart Association (NYHA) classification, and quality of life at 6 months. Rate of BP control were under controlled will be compared between the two groups, which is defined as SBP <140 mmHg and diastolic BP <90 mmHg at 6 months. In addition, drug adherence and changes in echocardiographic parameters, including left atrial volume index, left ventricular mass index, E/e', e', pulmonary artery systolic pressure, and GLS will be assessed between the two groups. All adverse reactions and the incidence of the following adverse events of special interest during treatment will be assessed: symptomatic hypotension and bradycardia (investigator-reported).

**Table 4:** Primary and secondary endpoints.

	Endpoints detail
Primary endpoint	Time-averaged proportional change in NT-proBNP concentration from baseline through months 3 and 6
Secondary endpoints	Proportion of patients with NT-proBNP decrease >20% from baseline through months 3 and 6
	Composite of all-cause mortality and all-cause hospitalization at 6 months
	Change in blood pressure and heart rate at 6 months
	Rate of blood pressure control at 6 months
	Change in NYHA classification at 6 months
	Change in quality of life assessed by visual analog scale (0–10)
	Drug adherence at 6 months
	Changes in echocardiographic parameter including LAVI, LVMI, E/e’, e’, pulmonary artery systolic pressure and GLS at 6 months

GLS, global longitudinal strain; LAVI, left atrial volume index; LVMI, left ventricular mass index; NT-proBNP, N-terminal pro B-type natriuretic peptide; NYHA, New York Heart Association.

Blood sampling tests at the screening visit will be conducted by the laboratories at each participating institute, which are certified by the Korean Association of Quality Assurance for Clinical Laboratory. Measurement of NT-proBNP by an electro-chemiluminescence immunoassay method using cobas® 8,000 (Roche Diagnostics, Mannheim, Germany) will be performed in a central laboratory at randomization and at 6 months (visit 4). Quality of life will be assessed using a visual analog scale ranging from 0 to 10, with 0 being the worst and 10 being best. Drug adherence will be assessed by “pill count” measurements. The patients will bring the remaining tables to each scheduled visit, and trained and certified researchers will count the number of returned drugs and calculate drug adherence as follows:

$$\text{Drug adherence }=\frac{\text{number of pills dispensed-number of pills returned}}{\text{number of days between dispensing date and follow-up date}}$$

Baseline echocardiographic data collected within 2 months before randomization will be used. All images will be obtained using a standard ultrasound system with 2.5 MHz probes manufactured by GE, Phillips, and Siemens. Standard techniques will be used to obtain the M-mode, two-dimensional, and Doppler measurements. The LV dimensions, LVEF, and other echocardiographic parameters will be obtained according to the guidelines of American Society of Echocardiography and the European Association of Cardiovascular Imaging (32). Echocardiographic parameters at each institution will be archived in the Digital Imaging and Communications in Medicine format. GLS will be evaluated by a central echocardiography laboratory at the Samsung Medical Center, Seoul, Korea. GLS will be assessed using two-dimensional speckle-tracking echocardiography (2D-STE) on index echocardiography (33). The 2D-STE data will be analyzed using TomTec software (Image Arena 4.6, Munich, Germany) for deformation analyses (2-dimensional cardiac performance analysis) (27). For deformation analysis, endocardial borders are traced on the end-systolic frame in three apical views (4-, 2-, and 3-chamber), with end-systole defined by the QRS complex or as the smallest LV volume during the cardiac cycle. The software tracks speckles along the endocardial border and myocardium throughout the cardiac cycle. The peak longitudinal strain is computed automatically, generating regional data from six segments (anterior, anteroseptal, anterolateral, inferior, inferoseptal, and inferolateral) and an average value for each view. The GLS is determined as the average peak longitudinal strain of 18 LV segments from standard apical views. For patients with SR, analyses will be performed on a single cardiac cycle; for patients with AF, the strain values will be calculated as the average of three cardiac cycles. All strain measurements will be performed by strain specialists who are blinded to other data of each patient. All strain analyses will be performed by a single investigator. In our previous study, intra-observer variability in GLS was assessed in 30 randomly selected patients (34). The coefficient of variation has been identified as 5.8% for the GLS, and the intraclass correlation coefficient was 0.95 for GLS [95% confidence interval (CI), 0.91–0.98].

### Sample size and statistical analysis

To date, no previous study has evaluated the effects of carvedilol-SR on HFpEF. Due to the absence of prior research and the pioneering nature of our intervention, a sample size calculation was not conducted at this preliminary stage. As this was a pilot study to explore the benefit of carvedilol-SR for HFpEF, we decided to enroll a total of 100 participants (50 in the carvedilol-SR group and 50 in the control group) across the three institutes, considering the duration of the study and the number of participating hospitals.

Categorical variables will be reported as frequencies (percentages), and continuous variables will be expressed as means ± standard deviations or medians with interquartile ranges. Categorical variables will be compared using Pearson's chi-square test or Fisher's exact test, and continuous variables will be compared using Student's *t*-test or the Mann–Whitney *U*-test.

Primary analysis of the proportional change in NT-proBNP concentration from baseline on a logarithmic scale will be performed using an analysis of covariance model with an adjustment for the baseline value. This will be calculated as the average of the geometric means of NT-proBNP at months 3 and 6 divided by the geometric mean of NT-proBNP at baseline in a natural logarithmic scale (i.e., ratio of geometric means) and summarized as the difference between the treatment groups in the ratio of the geometric means. Cumulative clinical-event rates will be calculated according to the Kaplan–Meier method, and the differences in clinical outcomes between the two treatment groups will be assessed using the log-rank test. Hazard ratios and associated 95% CIs will be calculated using a Cox proportional-hazards model. The incidence of adverse events will be calculated as relative risks with associated 95% CIs.

Missing values due to unevaluable samples or early study discontinuation will not be imputed. Patients with missing baseline NT-proBNP and/or missing 3 and 6 months will not be included in the primary analysis. The data will be primarily analyzed using intention-to-treat analysis, including all randomized patients. We will also perform a per-protocol analysis, which include all randomized patients who receive at least one dose of the study drug during the double-blind period and have no major protocol deviations. Safety outcomes will be analyzed in the safety set of patients who received the trial drug at least once.

Primarily, we will assess the efficacy of carvedilol-SR on the reduction of NT-proBNP in HFpEF compared with a placebo. In addition, we will evaluate the differential effects of carvedilol-SR on primary outcomes according to prespecified variables, including baseline GLS, and NT-proBNP, LVEF. We planned to use the cutoff value of 14% GLS, taking into account a previous study (28), and also planned to use the median cutoff value of the GLS.

All tests will be two-tailed, and a *P*-value <0.05 will be considered statistically significant. Statistical analyses will be performed using R version 4.2.3 (The R Foundation for Statistical Computing, Vienna, Austria).

## Discussion

This randomized controlled trial will investigate the efficacy and safety of carvedilol-SR in reducing NT-proBNP levels in patients with HFpEF and hypertension. We believe that this prospective double-blind multicenter trial will be particularly helpful in evaluating the differential effects of these drugs on HFpEF according to GLS.

The effects of beta-blockers on patients with HFpEF remain controversial. The SENIORS trial sub-analysis showed a trend towards a beneficial effect of nebivolol on HF outcomes in patients with LVEF >35% (12). However, the proportion of patients with LVEF ≥50% was not high (only 15%), limiting the ability to draw conclusions about HFpEF. Recently, in a HFpEF analysis in the SwedeHF registry, β-blockers were not associated with a change in risk for HF admissions or cardiovascular deaths (17). In addition, in the largest propensity score-adjusted cohort of 435,897 patients with HF and EF ≥40%, beta-blocker use was associated with a higher risk of HF hospitalization as EF increased (18). Potential benefits were observed in patients with HF with mildly reduced EF, while potential risks were observed in patients with higher EF (particularly >60%). Although a more recent meta-analysis showed that beta-blocker therapy reduced all-cause mortality in patients with HFpEF (15), overall, the results of studies on the benefits of beta-blockers in HFpEF are inconsistent, and LVEF may be an important predictor of response to beta-blockers in HFpEF. Moreover, hypertension, atrial fibrillation, and coronary artery disease are common comorbidities that may benefit from beta-blockers in patients with HFpEF. Based on previous studies, we designed a trial of carvedilol SR in patients with HFpEF and hypertension. Considering only a few randomized clinical trials were performed and the LVEF cut-off value was different among studies (12–14), we will enroll patients with LVEF ≥50% and hypertension, regardless of atrial fibrillation or coronary artery disease.

Several drugs that have been shown to be effective in HFrEF have failed to reduce mortality in patients with HFpEF (20, 35–37). Because HFpEF is a heterogeneous and complex syndrome rather than a single disease entity, medical therapy can be challenging, and only specific phenotypes may respond to a particular therapeutic intervention (7, 38). Given the clinical importance of identifying a group for whom beta-blockers are effective in HFpEF, we assumed that GLS could be used to identify patients with HFpEF who would benefit from these drugs. The previous study showed that beta-blockers were associated with improved survival in patients with low GLS (GLS <14%) but not in those with GLS ≥14% among patients with LVEF of ≥40% (28). Although this previous study differs from ours in that it included patients with HF who had mildly reduced ejection fraction (LVEF 41%–49%) and focused on 5-year mortality, which exceeds that of the present study, we hypothesized that patients with HFpEF and reduced GLS might benefit similarly from beta blockers, like those with HFrEF, among whom most patients have reduced GLS. Therefore, we will measure the GLS at the central echocardiography laboratory and analyze the differential effect of carvedilol-SR on change in NT-proBNP level according to the GLS categories. We will use the cutoff value of 14% GLS based on a previous study (28), and also plan to use the median cutoff value of the GLS.

The mechanism by which beta-blockers may confer benefits in HFpEF is not clearly understood. The main potential mechanism includes sympathetic antagonism of beta-blockers with the potential benefits of lowering BP and R, enhancing relaxation, increasing diastolic filling, improving ventricular remodeling, decreasing myocardial oxygen demand, and lowering arrhythmic threshold (16, 23). Moreover, beta-blockers might confer benefit in HFpEF through improving metabolic activity and endothelial inflammation. Because the effectiveness of beta-blockers in HFpEF has not been clearly demonstrated in large trials and the mechanisms are not well understood, these limitations led us to conduct a pilot randomized controlled trial to investigate the effectiveness of beta-blockers in HFpEF.

There are at least 20 commercially available beta-blockers for clinical use. Although beta-blockers share common mechanisms, they differ in their specific activities, particularly in their selectivity for the adrenergic receptors (16, 39). Carvedilol, one of the third-generation beta-blockers, has a nonselective alpha-1, beta-1, and beta-2 adrenergic activities and has been extensively studied in patients with HFrEF (8, 29). Some studies have shown that carvedilol improve diastolic dysfunction, cardiac remodeling, or NYHA functional class in HFpEF (40–42). Although we presume that there might be a class effect of beta-blockers in HFpEF rather than a specific drug effect, we decided to evaluate the effect of carvedilol-SR in HFpEF, taking into account data from these previous studies.

Many patients with HF are older and have multiple comorbidities; therefore, they take multiple medications (1). Complex medication regimens lead to poor medication adherence and poor clinical outcomes (43). In addition, the high levels of medication adherence observed in well-controlled clinical trials may differ from those observed in real-world clinical practice. To improve drug compliance with carvedilol, a longer-acting carvedilol-SR has been developed and is known to maintain an effective plasma concentration without exceeding the critical value for adverse effects (30). Carvedilol-SR has the advantage of being administered only once a day and is non-inferior to standard carvedilol-IR administered twice a day in patients with HFrEF (44). Therefore, we used long-acting carvedilol-SR instead of carvedilol-IR in this study population with HFpEF and hypertension, which may represent a potential strength.

### Strengths and limitations

This study had several limitations. First, it will only include East Asian patients, raising concerns about the extrapolation of these results to other ethnicities. Second, the number of participants to be enrolled in this study is not large, and it has a pilot study perspective. Third, the carvedilol dose will be up-titrated at the discretion of the treating physician and not according to the standard protocol in this trial, which could be a confounding factor. Fourth, the primary endpoint, change in NT-proBNP levels is a surrogate marker and may be influenced by confounding factors, such as the usage or dose of diuretics, which may be unequally controlled between the two groups. A high body mass index may also affect the level of this biomarker. Fifth, there will be no objective measure of functional status in terms of the 6-minute walk test or maximal oxygen uptake from the cardiopulmonary exercise testing. Despite these limitations, a major strength is that this study is a multicenter, randomized, double-blinded controlled trial with a novel design to evaluate the efficacy of carvedilol-SR in patients with hypertension and HFpEF and to assess the differential effects of these drugs according to GLS.

## Ethics statement

The studies involving humans were approved by Seoul National University Bundang Hospital (IRB B-2003-601-002). The studies were conducted in accordance with the local legislation and institutional requirements. The participants provided their written informed consent to participate in this study.
